# The Evolutionary Dynamics of Genetic Mutational Load Throughout Tomato Domestication History

**DOI:** 10.1111/mec.70024

**Published:** 2025-07-15

**Authors:** Hamid Razifard, Sofia Visa, Naama Menda, Lukas Mueller, Denise Tieman, Esther van der Knaap, Ana L. Caicedo

**Affiliations:** ^1^ Institute for Applied Life Sciences University of Massachusetts Amherst Amherst Massachusetts USA; ^2^ Department of Mathematics and Computer Science College of Wooster Wooster Ohio USA; ^3^ Boyce Thompson Institute Ithaca New York USA; ^4^ Horticultural Sciences, Plant Innovation Center University of Florida Gainesville Florida USA; ^5^ Department of Horticulture University of Georgia Athens Georgia USA; ^6^ Institute of Plant Breeding, Genetics and Genomics University of Georgia Athens Georgia USA; ^7^ Center for Applied Genetic Technology University of Georgia Athens Georgia USA; ^8^ Department of Biology University of Massachusetts Amherst Amherst Massachusetts USA

**Keywords:** cost of domestication, deleterious alleles, mutational load, solanum, tomato

## Abstract

Understanding the impact of domestication on deleterious mutations has fascinated evolutionary biologists and breeders alike. A ‘cost of domestication’ has been reported for some organisms through accumulation of gene disruptions or radical amino acid changes. However, recent evidence paints a more complex picture of this phenomenon in different domesticated species. In this study, we used genomic sequences of 253 tomato accessions to investigate the evolution of deleterious mutations and genomic structural variants (SVs) through tomato domestication history. We apply phylogeny‐based methods to identify deleterious mutations in the domesticated tomato as well as its semi‐wild and wild relatives. Our results implicate a downward trend throughout domestication in the number of genetic variants, regardless of their functional impact. This suggests that demographic factors have reduced overall genetic diversity, leading to lower deleterious load and SVs as well as loss of some beneficial alleles during tomato domestication. However, we detected an increase in proportions of nonsynonymous and deleterious alleles (relative to synonymous and neutral nonsynonymous alleles, respectively) during the initial stage of tomato domestication in Ecuador. Additionally, deleterious alleles in the commonly cultivated tomato seem to be more frequent than expected under a neutral hypothesis of molecular evolution. Our analyses also revealed frequent deleterious alleles in several well‐studied tomato genes, probably involved in response to biotic and abiotic stress as well as fruit development and flavour regulation. To provide a practical guide for breeding experiments, we created TomDel, a public searchable database of 21,162 potentially deleterious alleles identified in this study (hosted on the Solanaceae Genomic Network; https://solgenomics.net/).

## 1 Introduction

Understanding the circumstances that allow proliferation of deleterious mutations in genomes is a fascinating evolutionary question and one of great relevance for breeding applications. The occurrence of deleterious mutations in crop genomes has the potential to reduce crop productivity, making the detection and removal of these mutations from breeding pools an important goal (Sun, Wang, Wei, et al. 2023; Dwivedi et al. 2023). Additionally, in some cases mutations that would be deleterious in the wild may in fact be beneficial in agricultural contexts, again suggesting value in detecting such mutations (Dwivedi et al. 2023). In recent decades, several studies have reported accumulation of deleterious mutations during domestication of both plants (e.g., Lu et al. 2006; Liu et al. 2017; Zhou et al. 2017) and animals (e.g., Schubert et al. 2014; Marsden et al. 2016), often referred to as a ‘cost of domestication’ (Moyers et al. 2018). A review of studies on deleterious mutations in two domesticated plants, soya bean and rice, as well as five domesticated animals, dog, pig, rabbit, chicken and silkworm (Makino et al. 2018), concluded that, except in pigs, those organisms showed elevated proportions of nonsynonymous mutations when compared with their wild relatives. The exception observed in pigs was attributed to absence of strong bottleneck during pig domestication as well as possible gene flow with its wild ancestors. Similarly, a study on sunflower (Renaut and Rieseberg 2015) revealed a higher proportion of deleterious alleles in the domesticated sunflower, although the absolute number of deleterious mutations was similar to that of its wild ancestor. In tomato, a prior study has implied accumulation of deleterious mutations during domestication, based on a transcriptomic comparison between domesticated and wild tomato species (Koenig et al. 2013).

A comprehensive understanding of the evolution of deleterious mutations through domestication requires population‐wide genomic surveys based on comparisons between domesticated organisms and their closely related wild and semi‐domesticated populations. Adopting such an approach, more recent studies have revealed complexities in the evolutionary patterns of deleterious mutations in a small number of domesticated organisms. For example, estimates of mutational load, calculated as the sum of derived deleterious alleles, were higher in domesticated maize compared to its wild relative, but this same pattern was not observed for domesticated sorghum, perhaps due to its transition to a selfing mating system (Lozano et al. 2021). Therefore, the prevalence of deleterious mutations might differ even between closely related species. Similar to sorghum, domesticated soya bean was recently reported to harbour fewer deleterious alleles than its wild relatives (Kim et al. 2021), although prior studies had implied the opposite pattern (Lam et al. 2010). These conflicting conclusions point to a major difficulty in cost‐of‐domestication studies, which is a lack of consensus on the definition and methods used to evaluate deleterious alleles.

The mutational load, defined as the reduction in fitness due to harmful mutations (Kimura 1983), is largely shaped by the cumulative count of deleterious mutations occurring in a genome (see Moyers et al. 2018; and references therein). The mutational load present in a population is shaped by a unique combination of demographic and genomic factors. One of the major demographic processes impacting deleterious mutations through domestication is the reduction in the effective population size due to bottleneck events, which leads to more genetic drift and less efficient selection (Gaut et al. 2018), thus allowing deleterious alleles to persist. This is more evident in populations that have undergone a recent bottleneck event. However, sustained bottlenecks can lead to the removal of deleterious mutations, likely through the purging of highly deleterious alleles (Gaut et al. 2015; Grossen et al. 2020; Bortoluzzi et al. 2020). Additionally, a transition to an inbreeding mating system, which is a common phenomenon among domesticated organisms (e.g., Vallebueno‐Estrada et al. 2016; Cutter 2019), can more efficiently remove recessive deleterious mutations from the population by exposing them to selection through an increase in homozygosity (Byers and Waller 1999; Liu et al. 2017; Dwivedi et al. 2023).

Other demographic processes can affect the mutational load during domestication. For example, domesticated organisms can often experience rapid population growth as humans expand their cultivation in new geographic regions, which can lead to the accumulation of new deleterious mutations through ‘gene surfing’ when drift acts on the expanding edges of the population (Klopfstein et al. 2006; Travis et al. 2007). Asexual reproduction via clonal propagation can also lead to accumulation of deleterious mutations due to lack of crossing‐over. This phenomenon is often referred to as ‘Muller's ratchet’ (Muller 1932) and has been reported in plants, for example, cassava (Ramu et al. 2017), animals, for example, Bdelloid rotifers (Barraclough et al. 2007) and bacteria, for example, *Escherichia coli* (Elena and Lenski 1997).

Among genomic factors influencing the evolution of deleterious mutations, genomic structural variants (SVs) can disrupt genes, which might negatively affect the fitness of an organism (reviewed by Yuan et al. 2021). With the rapid advances in genomic research in recent years, several studies have revealed valuable insights on the prevalence of SVs in crop species, for example, *Brassica napus* L. (Chawla et al. 2021), rice (Fuentes et al. 2019), grapevine (Zhou et al. 2019), soya bean (McHale et al. 2012; Anderson et al. 2014), maize (Mahmoud et al. 2020); (Yang et al. 2019) and tomato (Wang et al. 2020; Alonge et al. 2020; Domínguez et al. 2020). However, the relationship between SVs and deleterious mutations has rarely been addressed. Notably, a study by Hämälä et al. (2021) on the cacao tree ( *Theobroma cacao* ) revealed higher numbers of potentially deleterious SNPs in genomic regions impacted by SVs.

The degree of genetic linkage (infrequent crossing‐over between genomic regions with smaller physical distance or due to other variations in recombination rate) is also an important factor in the prevalence of deleterious mutations in domesticated organisms. Due to linkage, artificial selection on favourable traits in the organisms under domestication can lead to the accumulation of deleterious mutations within or near genomic regions experiencing selection; this phenomenon is referred to as ‘genetic hitchhiking’ (Smith and Haigh 1974; Barton 1995).

Considering the complex interactions of demographic and genomic factors operating on a population, a robust analysis of mutational load in any domesticated species must be placed within the unique context of the domestication history of that organism. Currently available methods for detecting deleterious mutations (reviewed in Moyers et al. 2018) include consideration of the predicted impact of sequence changes in functionally annotated regions, but more often involve comparing protein sequences from an organism of interest and the orthologous protein sequences from closely related species (Ng and Henikoff 2003; Choi et al. 2012; Kono et al. 2016). The underlying assumption of these methods is that amino acid changes in positions that are highly conserved phylogenetically are considered to be deleterious.

To date, population‐level insight on deleterious mutations, especially in the intermediate stages of domestication between wild and domesticated populations, is lacking for many crops, including the commonly cultivated tomato. Insights on the evolution of deleterious mutations through tomato domestication could have immense economic value considering tomato's status as the world's most valuable vegetable crop (United Nations Food and Agriculture Organization 2013). The commonly cultivated tomato has a complex domestication history, with several intermediate populations occurring in Latin America (Blanca et al. 2015; Razifard et al. 2020). These populations have experienced different demographic changes. Thus, a thorough analysis of deleterious load in tomato entails special attention to population genomic factors affecting deleterious load in these intermediate stages as well as the functions of genes impacted by deleterious mutations.

In this study, we investigate the evolution of deleterious mutations through tomato domestication history, focusing on the evolution of deleterious mutations in the context of population genomic factors. Specifically, we used population genomic tools to (a) create novel insights on mutational load in the domesticated tomato and its closely related populations, (b) track the trajectories of deleterious alleles within wild, semi‐domesticated and domesticated tomato populations, (c) illuminate potential correlation between SVs and deleterious mutations and (d) create a functional link between deleterious mutations in the commonly cultivated tomato and some phenotypes of interest for breeding purposes.

## 2 Materials and Methods

### 2.1 Plant Material and Genotype Data

A dataset of genome‐wide variants created in a previous study (Razifard et al. 2020) was used to identify deleterious mutations in wild and domesticated tomatoes. The input dataset included 295 accessions of diverse SP ( *Solanum pimpinellifolium* ) and SLC ( *S. lycopersicum var. cerasiforme* ) from South America, SLL ( *S. lycopersicum var. lycopersicum* ) from the Americas as well as improved material from outside of the Americas, and one accession of *Solanum pennellii* L. (EA00585), serving as outgroup. All plant material was sourced from germplasm banks and was self‐fertilised for two generations for seed amplification prior to the Razifard et al. (2020) study. The reference genome used for calling variants was ‘Heinz 1706’, build SL2.50 (Tomato Genome Consortium 2012). From the input dataset, we excluded accessions with > 25% missing data as well as sites with insertion‐deletions (indels), leaving 15,011,193 SNPs in the final dataset.

The missing genotypes in the final dataset were imputed using LinkImpute version 1.1.4 (Money et al. 2015), with the default settings (‘LD‐kNNi’, imputation of missing genotypes based on k‐nearest neighbours as well as linkage disequilibrium). The resulting imputed VCF was used in the initial analyses (TreeMix and SIFT) described in the following sections. The outgroup accession was used to determine the ancestral alleles for all SNPs in the final dataset, that is, considering the allele observed in the outgroup as ancestral and the allele that differed from the outgroup as derived.

### 2.2 Group Delimitations

The main population was defined in a mostly consistent manner with the phylogenetic relationships provided in Razifard et al. (2020) with the following exceptions. Due to a low number of individuals for some populations of SP and to more clearly capture the changes after the divergence of other tomato groups from SP, we combined all three SP populations – a population of SP from northern Ecuador (SP NECU), one from southern Ecuador (SP SECU) and one from Peru (SP PER) – into a single group. We also split the cultivated tomato group (SLL) into two major populations: a clade of SLL accessions from the Americas (‘SLL Americas’) and another clade comprising mostly modern SLL accessions from outside the Americas (‘SLL modern’), to better distinguish the effects of tomato domestication from later improvement.

### 2.3 Population‐Level Analyses

We calculated nucleotide diversity (*π*) and Tajima's *D* for each population using VCFtools within 10‐kb windows. For estimating both *π* and Tajima's *D*, we incorporated a filter of minimum allele frequency (MAF) < 0.001 to exclude potentially erroneous rare variant calls. It is noteworthy that *π* calculations from VCFtools have recently been shown to have downward bias in genomic windows with a high proportion of missing genotype calls, which are classified as invariant instead of missing (Korunes and Samuk 2021). For SNPs, we found very few genomic regions (0.08%–4% of windows) that contained a high proportion (> 25%) of missing genotype calls at the individual level. However, the reported values of *π* for our data do not distinguish between invariant and missing sites within each window. Thus, *π* may be underestimated in windows with poor sequencing depth, low genotyping quality and/or highly repetitive regions. A maximum‐likelihood population tree was constructed using TreeMix (Pickrell and Pritchard 2012). To account for the effect of linkage disequilibrium, stretches of 1200 SNPs were grouped in separate windows (‘‐k 1200’), representing ~10‐Mb windows in the tomato genome (Pickrell and Pritchard 2012).

### 2.4 Identification of SNP Categories and Evaluation of Mutational Impacts

Two methods were used to classify SNPs into different categories and predict the impact of derived alleles. First, all sites were evaluated using SIFT (Ng and Henikoff 2003; Kumar et al. 2009), which categorised SNPs as non‐coding, synonymous and nonsynonymous; the effect of nonsynonymous SNPs was further predicted as ‘deleterious’ or ‘deleterious (low confidence)’. Second, following the procedure of Renaut and Rieseberg (2015), SNPs identified as nonsynonymous by SIFT were further evaluated using PROVEAN (Choi et al. 2012). SNPs with PROVEAN score < −2.5 were considered potentially deleterious, as suggested by Choi et al. (2012). Similar to other tools used for assessing variant impacts, SIFT and PROVEAN assume that mutations occurring at evolutionarily conserved coding regions of the genome are likely to be deleterious. However, the programmes use different mathematical models for calculating a score for each variant. SIFT computes a score based on the frequency of observed amino acids in the protein coded by a certain gene, across the gene phylogeny, as well as the distribution of unobserved amino acids based on a Dirichlet mixture model. PROVEAN provides a score for each variant based on the similarity of each protein sequence to a functional homologue and the change in similarity caused by incorporating each amino acid change to that protein. According to the PROVEAN model, amino acid changes that make a protein sequence less similar to a functional homologue are expected to be deleterious.

In all downstream analyses, only SNPs identified as deleterious by both SIFT and PROVEAN were considered deleterious. Nonsynonymous SNPs not classified as deleterious were considered neutral.

To avoid complications due to reference bias, we conducted SIFT runs by substituting reference and alternate alleles with inferred ancestral and derived alleles, respectively. Also, we ran PROVEAN using predicted outgroup protein sequences as input, i.e., by modifying reference protein sequences to incorporate amino acids based on the alleles observed in the outgroup.

We developed custom R scripts (v. 3.6.3; http://www.R‐project.org/) to calculate the number of sites with derived alleles from each functional category (non‐coding, synonymous, neutral nonsynonymous and deleterious nonsynonymous) in each genome. To adjust for heterozygosity, the counts of heterozygous sites belonging to each category were divided by two.

### 2.5 Identification of Genomic Structural Variants (SVs)

Genomic structural variants (SVs) were identified for all accessions included in this study, using three independent pipelines, Lumpy, v. 0.3.0 (Layer et al. 2014), Delly, v. 0.8.7 (Rausch et al. 2012) and Manta, v. 1.6.0 (Chen et al. 2016), using default options. Among different SV types, insertions are often difficult to reliably detect using the currently available pipelines; therefore, we excluded insertions (only 9 in total) from our analyses. The output from all three pipelines was merged using SURVIVOR, v.1.0.7 (Jeffares et al. 2017) keeping SVs present in the outputs from at least two out of three pipelines, with a minimum size of 30 bp long. SVs in different accessions but on the same chromosome, with the same SV type, within 100 bp of one another and with similar length (90% overlap) were considered as the same SV.

For comparing SV results obtained from short‐ versus long‐read data, we conducted SV calling using both long‐read PacBio as well as short‐read Illumina data on LA2093, an SP accession sequenced by Wang et al. (2020). We performed long‐read SV calling using *pbsv* in *pacbio* software suite (https://github.com/PacificBiosciences/pbsv) and short‐read SV calling following the methods described above.

To avoid false‐positive identification due to missing data, rare SVs with minimum allele frequency (MAF) < 0.01 were also excluded from downstream analyses. All SVs were polarised by considering the genotype observed in the outgroup, *S. pennellii* , as the putative ancestral state.

A custom R script (available from https://github.com/hrazif/scripts_for_tomato_deleterious_mutations_paper) was developed to count the number of deleterious and neutral nonsynonymous mutations that overlap with different SV types in each accession. Another R script was developed for counting the number of SVs occurring in genic and non‐genic regions.

### 2.6 TomDel

We created a visual database of all deleterious alleles identified in this study within modern tomato and its closely related populations. To create this database, we used a custom R script (available from our GitHub repository) to plot genotype frequencies of all the SNPs with deleterious alleles in all the populations. The database is publicly available on GitHub (https://github.com/hrazif/TomDel‐0.1) and is also hosted on the Solanaceae Genomic Network (https://solgenomics.net/).

### 2.7 Private Alleles

We created a custom R script (available from GitHub repository) to prepare separate lists of derived deleterious mutations and the derived neutral nonsynonymous mutations that were unique to each of the populations. We defined private alleles as those that are present in only one population (derived allele frequency > 0) but absent in all other populations (derived allele frequency = 0). Counting the number of private alleles in each accession, as well as the follow‐up plotting and statistical comparisons, were conducted in R.

### 2.8 Statistical Comparisons

All statistical comparisons were conducted in R. The Kruskal–Wallis tests were used to determine whether populations have a significant effect on the distribution of the ratios of different types of mutations (e.g., derived deleterious/neutral nonsynonymous, referred to hereafter as *del./neutr. nonsyn*.). Then, Dunn's test was used to perform a pairwise comparison between the distributions of values between populations. The comparison between private and shared *del./neutr. nonsyn*. was based on a *T*‐test. A Kolmogorov–Smirnov test was conducted to compare the distribution of derived allele frequencies of deleterious and neutral nonsynonymous alleles. In all statistical tests involving multiple testing herein, the *p*‐value significance cut‐off was decided at 0.05, after adjusting for multiple testing based on Bonferroni correction.

### 2.9 Correlation Between Relative Mutational Load and Other Genomic Features

We explored potential correlation between *del./neutr. nonsyn*. and three genomic statistics: *π* (using VCFtools), recombination rate (based on the genetic map ITAG2.3 from www.solgenomics.net) and SweeD Likelihood ratio (testing for deviation of site frequency spectrum from neutrality; Pavlidis et al. 2013). Each statistic was calculated within 100‐kb windows for each population (using the ‘grid’ option for SweeD). Correlation between the three statistics and *del./neutr. nonsyn*. was explored separately for each statistic as follows. Windows were sorted by each statistic and divided into ‘high’ and ‘low’ groups, placing windows with top 50% values in the ‘high’ group and vice versa. Wilcoxon tests were conducted separately for each statistic to test for difference in the distributions of relative mutational load across individuals in ‘high’ and ‘low’ groups in each population.

### 2.10 GO‐Term Enrichment Analyses

We conducted GO‐term enrichment analyses, using R package topGO, v.2.28.0 (Alexa et al. 2006) on SNPs with deleterious alleles present in modern cultivated tomato (deleterious derived allele frequency > 0). Gene Ontology (GO) terms and their respective genes were obtained from the Solgenomics website (https://solgenomics.net/ftp/genomes/Solanum_lycopersicum/annotation/ITAG2.4_release/). Multiple algorithms – ‘classic’, ‘elim’, ‘weight’, ‘weight01’, ‘lea’ and ‘parentchild’ – were tested for these analyses, and their results were explored for shared GO terms. Statistical significance was measured using Fisher's Exact Test, and the resulting *p*‐values were independently adjusted for multiple testing, according to the way in which each method treats GO‐term relationships (further described in topGO user manual).

### 2.11 Genome‐Wide Association Studies (GWAS)

Several tomato fruit volatiles were quantified as described in Tieman et al. (2017). Briefly, three plants from each accession were grown in a randomised manner in Florida. Volatiles were extracted from ripe fruits and were quantified using gas chromatography combined with mass spectrometry.

The genomic variant dataset used for GWAS included small indels and ‘chromosome‐zero’ SNPs (SNPs on genomic contigs with uncertain placement in the genome), but excluded SNPs with minor allele frequency < 0.05 and missing rate > 10%. Also, to avoid potential false GWAS hits due to strong population differentiation, SP accessions were removed from the GWAS input dataset. The GWAS results were explored for overlap with deleterious mutations. Phenotypes with distributions deviating significantly from normality based on Shapiro test (*p*‐value < 0.01) were normalised in R. GWAS was conducted in GEMMA (v 0.94.1) using a mixed linear model (Zhou and Stephens 2012). The associations were adjusted for population structure according to a genetic relatedness matrix created also using GEMMA. Due to concerns about loss of valuable associations by over‐adjusting, the genomic principal components were not included as covariates. *p*‐values based on likelihood ratio test (LRT) were used for determining significant correlations. A significance cut‐off was determined based on the effective number of independent SNPs calculated using the Genetic Type I error calculator (GEC, v0.2) (Li et al. 2012).

## 3 Results

### 3.1 Population History and Statistics

To investigate the dynamics of deleterious mutations through tomato domestication history, we used a dataset of 15,011,193 single nucleotide polymorphisms (SNPs) obtained from genome‐wide sequencing of a diverse set of tomato accessions. These accessions included the closest fully wild relative of the domesticated tomato, that is, *Solanum pimpinellifolium* L. (SP); the intermediate group between SP and the commonly cultivated tomato, that is, *S. lycopersicum* L. var. *cerasiforme* (SLC), from South America and Mesoamerica; landraces of the domesticated tomato, that is, *S. lycopersicum* L. var. *lycopersicum* (SLL), from the Americas as well as modern varieties grown worldwide; and one accession of *Solanum pennellii* L. as an outgroup.

A maximum‐likelihood population tree obtained using TreeMix (Figure S1), and the geographic distribution of the accessions from each group, recapitulate our current understanding of tomato domestication history in South America and Mesoamerica, which has been discussed in detail in Razifard et al. (2020). Briefly, the SLC are a highly diverse group both phenotypically and geographically, with SLC populations extending from South America to Central America and Mexico. Three SLC populations from South America (SLC ECU, SLC PER and SLC San Martin) are considered semi‐domesticated because they tend to display domesticated‐like phenotypes. Of these SLC groups, SLC from Ecuador was likely the first intermediate group of tomatoes to diverge from wild SP. Two ‘weedy’ SLC populations, one mostly distributed in Mexico (SLC MEX) and another with an extensive distribution ranging from Mexico to Central America and northern South America (SLC MEX‐CA‐NSA), are the northernmost extensions of SLC and tend to display some wild‐like phenotypes (e.g., small fruit size) similar to SP (Figure S1; Barnett et al. 2022). Finally, the closest relative of the commonly cultivated tomato (SLL) is SLC MEX.

**FIGURE 1:**
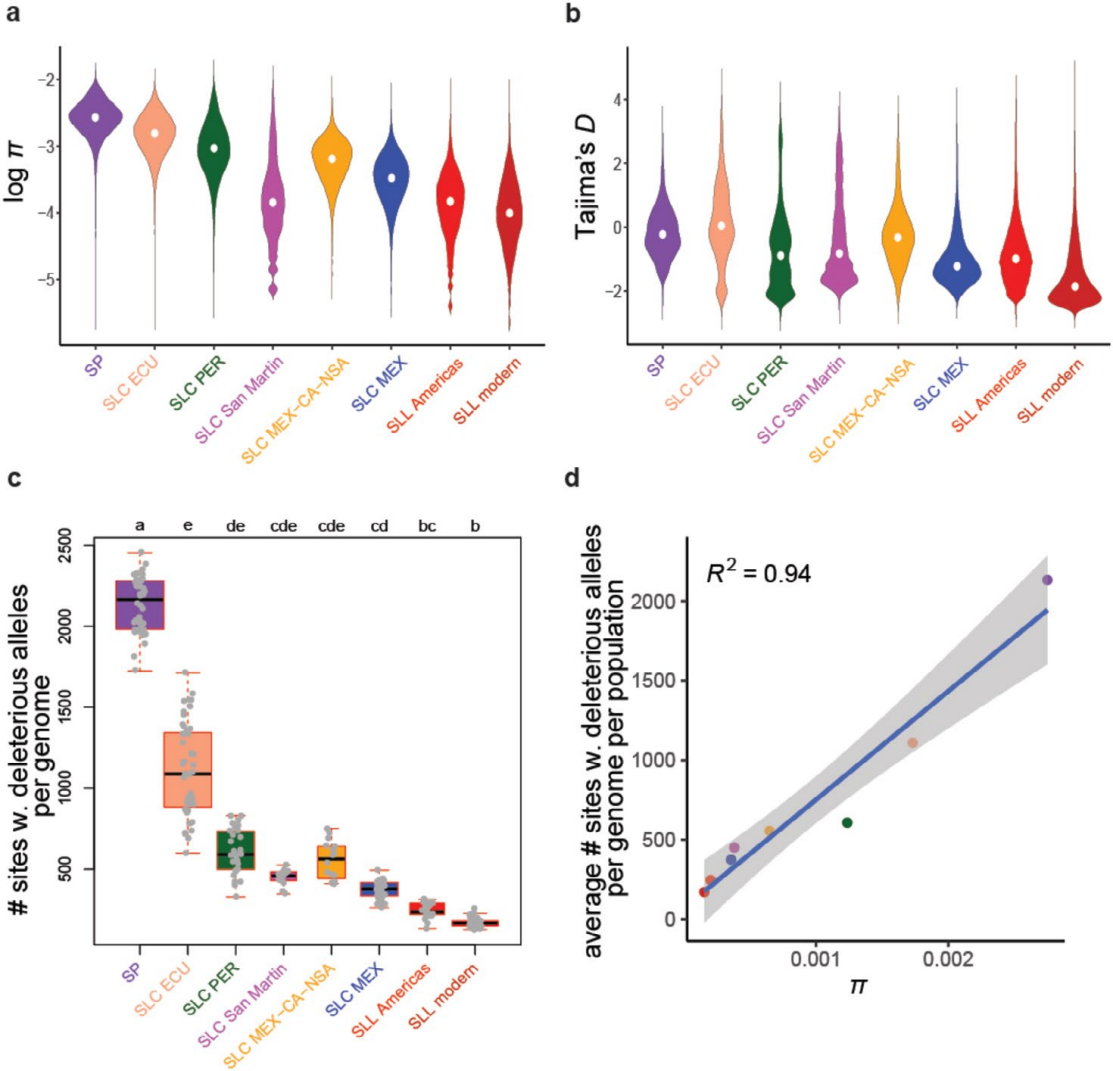
Tomato population statistics and mutational load. Nucleotide diversity, log *π*, (a) and evidence of selection, Tajima's *D*, (b) calculated for each accession within all tomato populations included in this study. All pairwise comparisons between populations are statistically significant for *π* and Tajima's *D* (*p*‐value < 2 × 10^−16^ based on Dunn's test). (c) Number of sites with derived alleles identified as deleterious by SIFT and PROVEAN in each of the tomato accessions within different populations. Results of pairwise statistical comparisons (Dunn's test) have been presented with lowercase letters; populations with a shared letter did not have a significant difference in their distribution of the number of sites with deleterious alleles. (d) A correlation analysis between *π* and the average number of deleterious alleles in each population. Point colours match population colours in (a–c).

To confirm patterns of genetic diversity and overall allele frequencies in the populations studied here, we estimated nucleotide diversity (π) and the deviation from a neutral allele frequency spectrum (Tajima's *D*) (Figure 1a,b). Notably, VCFtools is unable to distinguish missing from invariant sites, which can introduce a downward bias in the measurement of windowed π when structural variation is present among populations (Korunes and Samuk 2021). As a result, the estimates of π reported here may be lower and correlated with missingness in regions of the genome with a highly repetitive regions, poor mappability and/or low genotyping quality. However, there were few windows with high individual‐level missingness in our dataset (Table S6). While an abundance of rare alleles in SLL has been described previously (Razifard et al. 2020), results from this study show that it is ‘SLL modern’ – tomato accessions collected from outside the centres of tomato domestication in South America and Mesoamerica (Figure 1b) – that have the most negative median Tajima's *D*. Comparing median *π* between different populations shows a general downward trend during the transition from SP to SLC and from SLC to SLL, consistent with bottleneck events through tomato domestication history.

### 3.2 Assessing Mutational Load of Different Tomato Populations

To assess the occurrence of deleterious mutations among individual genomes in each tomato population, which we take as a measure of the mutational load, we explored the functional impact of each SNP. Reference bias could lead to both over‐estimation (false positive) as well as under‐estimation (false negative) of variants, especially when genomes under study are highly divergent (della Coletta et al. 2021). Therefore, it is unclear whether reference bias could lead to an overall change in the number of variants identified in a population in comparison to the reference genome. As an attempt to reduce the impact of reference bias and to focus more on novel alleles in tomato domestication history, we polarised all variants in all tomato populations studied here using a *Solanum pennellii* L. accession, then evaluated only derived alleles for their functional impact (detailed in Section 2).

A total of 134,446 SNPs were identified as containing nonsynonymous alleles by SIFT, of which 53,222 were identified as sites containing potentially deleterious alleles (‘deleterious’ or ‘deleterious, low confidence’). The SIFT list of nonsynonymous sites was provided as input to PROVEAN, which identified 36,583 sites as potentially deleterious. In the final list of deleterious alleles, we included 21,162 sites identified as potentially deleterious by both SIFT and PROVEAN.

We assessed the number of deleterious alleles carried by each genome and determined distributions in each population (Figure 1c), noting higher levels of mutational load in SP and decreased levels in SLL modern. We also compared the total number of derived alleles per genome belonging to different functional categories (non‐coding, synonymous, neutral nonsynonymous and deleterious nonsynonymous) between populations (Figure S2) and noted a similar trend as for mutational load. We thus evaluated the impact of nucleotide diversity on mutational load in each tomato population by conducting correlation analyses between nucleotide diversity and the average number of derived mutations belonging to the five different categories specified above (Figure 1d; Figure S2). These analyses reveal a significant correlation between nucleotide diversity and the number of derived mutations from all functional categories, regardless of the potential impacts of these mutations.

We considered that perhaps the drastic changes in genetic variation through domestication may be obscuring any patterns specific to deleterious mutations. We thus focused on the proportions of alleles from different mutational categories within each population, to detect any differences in the rates at which mutational loads have accumulated. Specifically, we looked at the ratio of the number of sites with derived nonsynonymous alleles to the number of sites with synonymous alleles (*nonsyn*.*/syn*.) per genome, as this measure has been used in other studies, as well as at the ‘relative mutational load’, here defined as the ratio of the number of sites with derived deleterious alleles to the number of sites with neutral nonsynonymous alleles (*del./neutr. nonsyn*.), which are nonsynonymous alleles not identified as deleterious by SIFT and PROVEAN (see Section 2). These ratio estimates are expected to be more robust to the effects of decreased diversity due to bottlenecks and inbreeding, than the absolute number of deleterious mutations.

**FIGURE 2:**
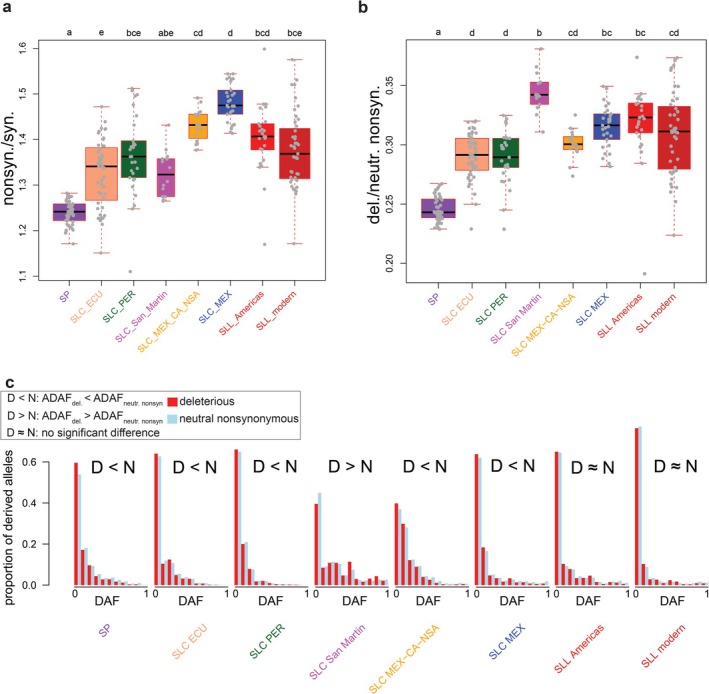
Prevalence of alleles with potentially high impact on fitness, compared with neutral alleles. (a) *nonsyn./syn*., the ratio of the number of sites with nonsynonymous alleles to the number of sites with synonymous alleles in each accession categorised within different tomato populations. (b) *del./neutr. nonsyn*., the ratio of the number of sites with deleterious alleles to the number of sites with neutral nonsynonymous alleles in each accession within different tomato populations. In both (a) and (b) results of a pairwise Dunn's test are shown with lowercase letters (see Figure 1 caption). (c) Site frequency spectra for derived deleterious (red) and derived neutral nonsynonymous alleles (blue) in each tomato population. For reach population, derived allele frequency (DAF) is shown in the range of 0 to 1, with bins of 0.1. Results of statistical tests (based on a Kolmogorov–Smirnov test) of average derived allele frequency of deleterious alleles (ADAF_del_) versus average derived allele frequency of neutral nonsynonymous alleles (ADAF_neutr.nonsyn._) are shown with D < N (ADAF_del_ is smaller than ADAF_neutr.nonsyn._), D > N (ADAF_del_ is greater than ADAF_neutr.nonsyn._ l) and D≈N (ADAF_del_ is not significantly different from ADAF_neutr.nonsyn._).

Per‐accession *nonsyn./syn*. (Figure 2a) varies widely between groups, but is significantly higher in SLC ECU, compared to SP. In contrast, there are no significant differences in *nonsyn./syn*. between SLC ECU and the other two South American SLC populations (SLC PER and SLC San Martin). SLC MEX‐CA‐NSA and SLC MEX, representing the northern populations of SLC, have higher *nonsyn./syn*. ratios, compared to SLC ECU. Conversely, a significantly lower *nonsyn./syn*. ratio in modern cultivated tomato (‘SLL modern’) is observed compared to SLC MEX, although the variance is large. However, no significant change in *nonsyn./syn*. is observed when comparing wild‐like SLC MEX and the SLL Americas.

While nonsynonymous substitutions have a higher probability of being deleterious than synonymous changes, the true mutational load in genomes is given by the proportion of nonsynonymous mutations deemed to be deleterious. We thus explored the patterns of per‐accession relative mutational load among and between all the populations included in this study (Figure 2b). Two major differences also appear when comparing the patterns of relative mutational load and *nonsyn./syn*. in the tomato population (Figure 2a,b). First, SLC MEX‐CA‐NSA does not have a significant difference in relative mutational load compared to SLC ECU, although its *nonsyn./syn*. ratio is significantly higher than in SLC ECU. Second, compared to SLC ECU, SLC San Martin harbours a significantly higher relative mutational load (Figure 2b), although its average *nonsyn./syn*. is not significantly different from SLC ECU. Finally, there is no significant difference in relative mutational load between the two SLL populations and their closely related populations, SLC MEX‐CA‐NSA and SLC MEX (Figure 2b), despite the lower *nonsyn./syn*. in modern SLL in comparison with SLC MEX (Figure 2a).

### 3.3 The Frequency Spectra of Deleterious Mutations in Tomato Populations

We further investigated the frequencies of derived neutral nonsynonymous and derived deleterious alleles in each tomato population (Figure 2c). In theory, deleterious alleles are expected to occur at low frequency in populations under drift‐selection equilibrium due to the action of background selection, which removes deleterious variants more readily than neutral variants. However, deleterious alleles that ‘hitchhike’ with positively selected variants can reach higher frequency in populations, and the reduced efficiency of selection in populations with small effective population size can also lead to higher‐than‐expected deleterious allele frequencies.

In most tomato populations studied here, the average derived allele frequency of deleterious alleles (ADAF_del_) is smaller than the average derived allele frequency of neutral nonsynonymous alleles (ADAF_neutr.nonsyn._) (Figure 2c). However, three populations deviated from this pattern: first, SLC San Martin has a higher ADAF_del_ than ADAF_neutr.nonsyn_. Second, the two SLL populations show no significant difference in ADAF_del_ versus ADAF_neutr.nonsyn._, indicating that the deleterious alleles that do exist in these populations are relatively common across accessions.

For each accession within a certain population, we also calculated the proportion of sites with derived deleterious alleles that are private to the population to which that accession belongs, that is, #Priv_del._/#All_del_. Similarly, we calculated #Priv_neutr.nonsyn._/#All_neutr.nonsyn._ for each individual (Figure S3). In all populations, except SP, a higher proportion of deleterious alleles than neutral nonsynonymous alleles were found to be private. This indicates that for SP deleterious alleles are as likely as neutral alleles to be shared with other populations.

We also estimated the average derived allele frequency for private alleles that are either deleterious or neutral nonsynonymous (Figure S4). Private deleterious alleles are as common as private neutral nonsynonymous alleles within each SLC and SLL population. In SP, however, private deleterious alleles had lower average frequency than observed for their private neutral counterparts.

Deleterious mutations are expected to accumulate near genomic regions under positive selection due to genetic hitchhiking via linkage disequilibrium (Moyers et al. 2018). Also, phylogenetically conserved regions of the genome are expected to harbour lower mutational load, due to the highly negative impact of deleterious mutations in such regions on the organism's fitness. To test these hypotheses and provide insights on the genomic factors affecting mutational load in tomato populations, we calculated relative mutational load in genomic windows of 100 kb for all the accessions. For each window, we also estimated recombination rate (*r*), nucleotide diversity (*π*) and signals of selective sweeps.

In all populations, genomic windows with higher nucleotide diversity have higher relative mutational load (Table 1). Also, in all populations, except SLC San Martin and the two SLL groups, windows with lower recombination rates, on average, have higher relative mutational load, consistent with recombination rates affecting the ability to purge deleterious mutations. However, in all populations, except SLL Americas, genomic regions with signals of selective sweeps (high *SweeD* likelihood ratio) have lower relative mutational load. In SLL Americas, we did not observe a statistically significant difference in relative mutational load between genomic regions with high and low signals of selective sweeps.

**TABLE 1:** A comparison of average relative mutational load, across accessions, in genomic windows with different levels of nucleotide diversity (*π*), recombination (*r*) and selective sweep signal (*SweeD*).

Population	Low *π*	High *π*	Low *r*	High *r*	Low *SweeD*	High *SweeD*
SP	0.62	0.84^s^	0.90	0.58^s^	1.07	0.40^s^
SLC ECU	0.50	0.74^s^	0.73	0.52^s^	0.89	0.35^s^
SLC PER	0.49	0.61^s^	0.64	0.45^s^	0.73	0.36^s^
SLC San Martin	0.40	0.46^s^	0.46	0.41^n^	0.50	0.37^s^
SLC MEX‐CA‐NSA	0.45	0.54^s^	0.55	0.44^s^	0.60	0.39^s^
SLC MEX	0.46	0.55^s^	0.55	0.47^s^	0.57	0.44^s^
SLL Americas	0.41	0.47^s^	0.47	0.41^n^	0.46	0.42^n^
SLL Modern	0.39	0.47^s^	0.45	0.41^n^	0.48	0.37^s^

*Note:* Results of Wilcoxon tests on windows with high and low average *π*, *r* and *SweeD* are shown with ‘s’ (significant) and ‘n’ (non‐significant).

### 3.4 Structural Variants and Their Impact on Mutational Load

To assess a potential association between deleterious alleles and genomic structural variants (SVs), we identified SVs based on short‐read data for all accessions. To further confirm the SVs identified using short‐read sequencing data, we conducted SV calling using both long‐read PacBio as well as short‐read Illumina data of LA2093, an SP accession sequenced in Wang et al. (2020). Our comparison revealed that the majority (69%) of SVs identified using short‐read sequencing data in LA2093 were also found using long‐read data (within 100 bp); thus, SVs identified in our population samples are likely to be highly reliable.

Using short‐read sequencing data for all accessions, we identified 1,809,695 SVs in our dataset, with a median length of SVs = 418 bp. However, many of these were of the same type and had a similar genomic position and length. Therefore, we merged the genotypes from such SVs, leaving 426,624 unique SVs. Further filtering of low‐frequency SVs (minimum allele frequency < 0.01) resulted in 97,654 SVs, which we used in the downstream analyses. We determined the derived SV alleles relative to the genotype observed in the outgroup, *S. pennellii* .

**FIGURE 3:**
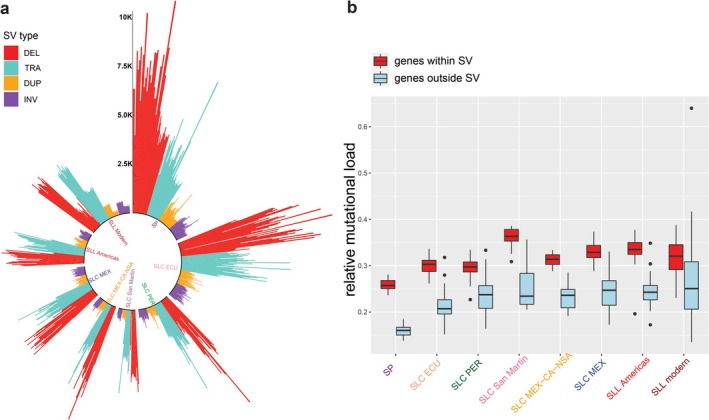
The evolution of structural variants (SVs) during tomato domestication and their association with mutational load. (a) The number of SVs from different categories (DEL, Deletions; DUP, Duplications; INV, Inversions; TRA, Translocations) identified in each accession belonging to different tomato groups. Each coloured bar represents one accession (b) A genome‐wide comparison between mutational load within and outside SV in each population. The comparisons within all populations are statistically significant (based a *T*‐test; *p*‐value < 0.001).

Our estimates of the number of derived SVs from different categories (deletions, translocations, duplications and inversions) were lower in the semi‐domesticated and domesticated tomato populations (Figure 3a). This is consistent with the pattern observed for deleterious mutations as well as mutations belonging to other categories (Figure 1c,d and Figure S2). We also conducted a comparison between genic and non‐genic regions of the tomato genome for the prevalence of SVs. We detected significantly more SVs in non‐genic regions (Figure S5), compared to genic regions (i.e., SVs within, containing or partially overlapping with genes, after adjustment for the size of genic versus non‐genic regions in the tomato genome). Among the tomato SVs in genic regions, most SVs were overlapping with introns and exons as opposed to UTRs (untranslated regions); however, the counts of SVs adjusted for the total sizes of intragenic features revealed an opposite pattern (Figure S6), with SVs more prevalent in the 5′ and UTRs of genes than in introns and exons and higher prevalence for SVs within exons than introns. Last, a comparison of levels of *del./neutr. nonsyn*. per genome in genes occurring in regions with and without SVs revealed that there was a significantly higher relative mutational load in genes within SV regions (Figure 3b; *T*‐test, *p*‐value < 0.001).

### 3.5 Deleterious Mutations of Interest for Tomato Breeding

To better understand the journeys of each deleterious allele throughout tomato domestication history, we estimated genotype frequencies of all deleterious alleles in modern tomato as well as its closely related populations. These results are provided in a publicly accessible repository named TomDel (available from https://github.com/hrazif/TomDel‐0.1, also hosted on the Solanaceae Genomic Network; https://solgenomics.net/). Genotype frequency plots for tomato genes can be searched with gene IDs, for example, Solyc01g005000, using the ‘Go to file’ option. On the Solanaceae Genomic Network, information and plots for deleterious mutations from each gene can be found by searching using the Search/Gene & Loci option; for example, see https://solgenomics.net/locus/9401/view for information on Solyc01g005000.

For some well‐studied tomato genes, we compared the proportions of deleterious genotypes between different tomato populations (Table S1), which revealed that deleterious alleles most often have become less frequent during the transition from SP to SLC, and many deleterious alleles are absent in SLL Modern.

We further explored our dataset for sites with deleterious alleles that are present in SLL Modern. These are often the alleles of highest interest for crop improvement. Of 21,162 sites with deleterious alleles in the entire dataset, 1677 sites contained deleterious alleles in modern SLL. A Gene Ontology (GO) term analysis on deleterious alleles present in modern SLL (Table S2) revealed mostly broad term such as ‘metabolic process’ and ‘oxidation–reduction process’, which implies that genes with a broad range of functions have been impacted by deleterious mutations. However, we also found more specific GO terms such as ‘inositol biosynthetic process’, related to ascorbic acid pathway (Calafiore et al. 2016) and ‘spermidine biosynthetic process’, related to pollen germination under heat stress (Song et al. 2002).

**FIGURE 4:**
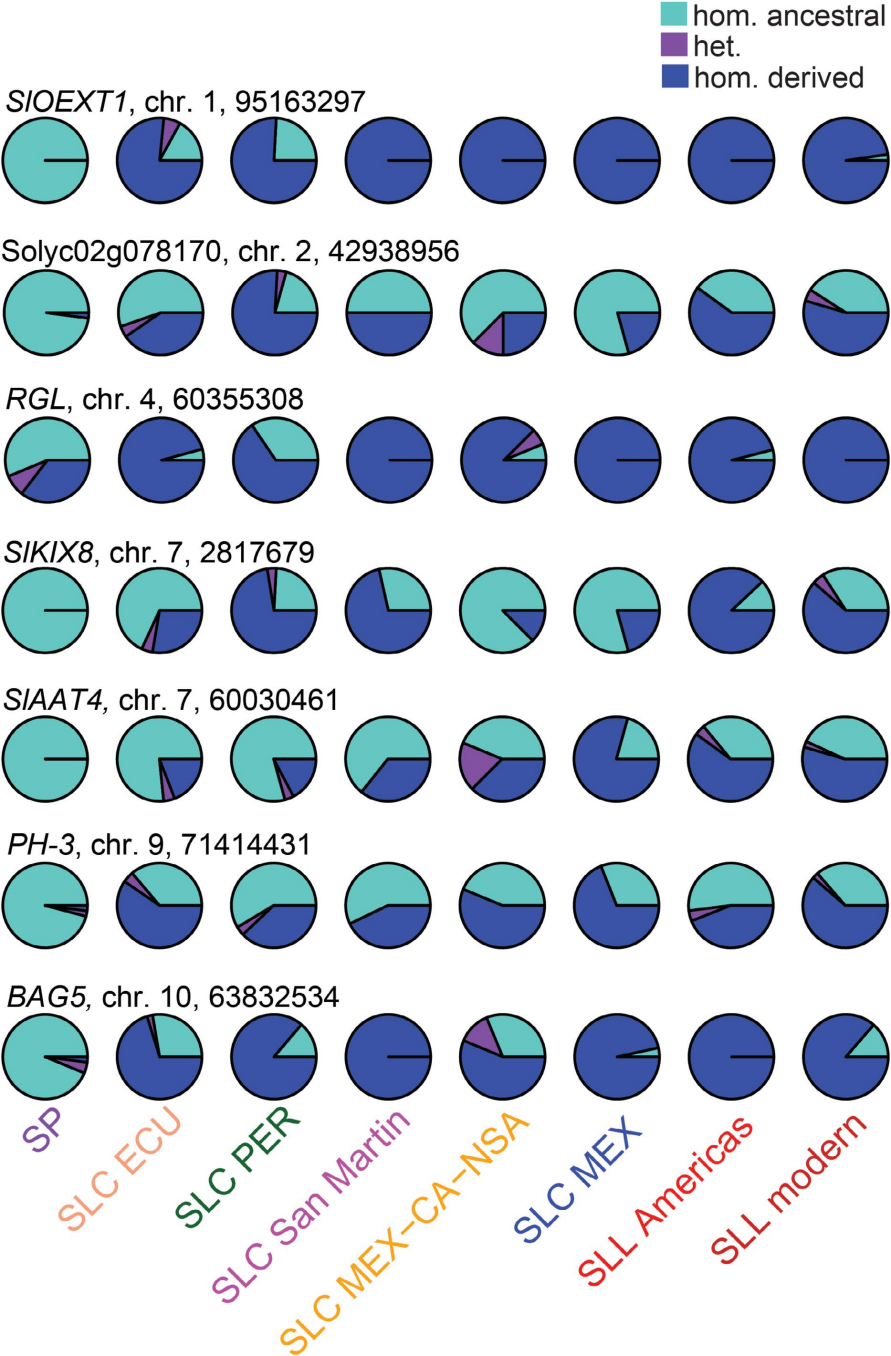
A population‐level comparison of genotype frequencies of some example sites with common deleterious alleles (allele frequency > 0.5) in modern SLL. These example sites represent those within well‐studied tomato genes or those with a known ortholog in other plants. Genotype frequencies are differentiated as follows: Homozygous derived deleterious in blue; homozygous ancestral in turquoise; and heterozygous in purple. The ancestral state is assumed to be the allele present in the outgroup, *S. pennellii* (see Section 2).

Among sites with deleterious alleles present in modern SLL, 184 sites had considerable deleterious genotype frequency (> 0.25), and in 103 sites, the deleterious homozygous genotypes were the common genotypes (frequency > 0.5; Tables S3 and S4). We detected genes with common deleterious genotypes involved in disease resistance, response to heat stress, fruit development and fruit flavour (Figure 4, Table 2). Notably, we found a common deleterious allele in *SlAAT4*, which might be involved in regulating tomato fruit flavour through the biosynthesis of fruit volatile esters.

**TABLE 2:** Examples of tomato genes with common deleterious genotypes (frequency > 50%) in SLL Modern.

Gene ID	Gene name	Gene function	References	Chr.	Del. pos.
Solyc01g107710	*SlOEXT1*	Extensin; cell wall biosynthesis and fruit ripening	Ding, Yang, et al. (2020)	1	95163297
Solyc02g078170	?	L‐type lectin receptor kinase; disease resistance	Wang et al. (2015)	2	42938956
Solyc04g074360	*RGL*	Rhamnogalacturonate lyase involved in fruit ripening	Tranchida‐Lombardo et al. (2018)	4	60355308
Solyc07g008100	*SlKIX8*	Kinase‐inducible domain interacting; leaf and fruit development	Swinnen et al. (2021)	7	2817679
Solyc07g049670	*SlAAT4*	Tomato alcohol acyltransferase; biosynthesis of fruit volatile esters	Goulet et al. (2015)	7	60030461
Solyc09g092280	*PH‐3*	Late blight resistance	Ren et al. (2019)	9	71414431
Solyc10g084170	*BAG5*	Chaperone regulator; response to heat stress	Ding, Mo, et al. (2020)	10	63832534

*Note:* The significance is shown using s (signficant) and n (non‐significant) for the ‘high’ group in each category.

Abbreviations: Chr., chromosome; Del. pos., position of the site with a deleterious allele.

**FIGURE 5:**
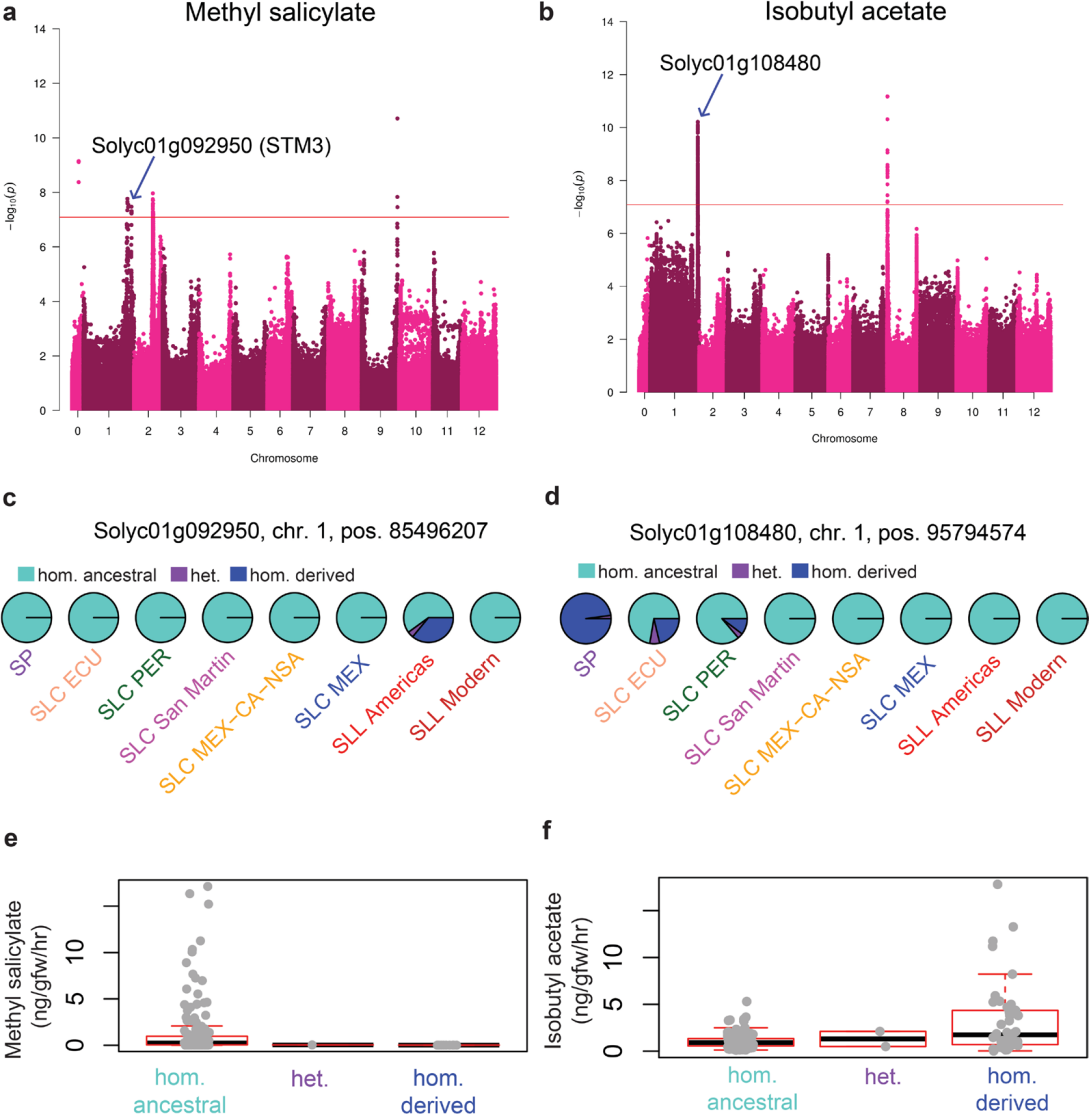
GWAS results on deleterious alleles associated with genes involved in tomato fruit volatile biosynthesis. Results are provided for methyl salicylate (a, c and e) and isobutyl acetate (b, d and f). For each volatile, Manhattan plots (a, b), population‐level comparisons of genotype frequencies (c, d) and their phenotypic distribution in different genotypes (e, f) are presented.

Considering the importance of fruit flavour genes for crop improvement, we further investigated the impact of deleterious mutations on fruit volatiles via genome‐wide association studies (GWAS) (Table S5). We discovered a deleterious allele significantly correlated with methyl salicylate content (Figure 5a). A deleterious allele on chromosome 1, position 85,496,207 (Figure 4a) is within Solyc01g092950 (STM3), which codes for a MADS‐box transcription factor. This deleterious allele was found only in SLL Americas and is negatively correlated with methyl salicylate levels (Figure 5c,e).

We also discovered a deleterious allele correlated with isobutyl acetate levels (Figure 5b). This deleterious allele on chromosome 1, position 95794574 (Figure 5b) is within Solyc01g108480, which codes for a serine carboxypeptidase. This deleterious allele is common in SP but rare in SLC ECU and SLC PER and absent in the remaining SLC populations (Figure 5d). Additionally, that allele has a positive correlation with isobutyl acetate (Figure 5f).

## 4 Discussion

Through this study, we have attempted to create a nuanced view of the evolution of deleterious mutations through tomato domestication history. To achieve this goal, we have taken a multi‐pronged approach, which involves studying deleterious mutations in the context of demographic and genomic changes, discussed below. We acknowledge that the methods we used detect mutations causing major changes in genes that are assumed to be potentially deleterious, but their true effect on fitness is likely to be dependent on environmental context. For ease of reading, we continue to refer to these alleles as ‘deleterious’ below but underscore that further studies would be needed to determine the true effect of each allele in wild and cultivated environments.

### 4.1 Evolution of Deleterious Mutations Through Tomato Domestication History

A ‘cost of domestication’ (Moyers et al. 2018) has often been assumed in the form of a higher accumulation of deleterious mutations in the genomes of domesticated crops and animals. It would be reasonable to predict such a domestication cost for tomato for several reasons. First, there has been an overall reduction in genetic diversity during tomato domestication history (Figure 1a), consistent with previous hypotheses on bottleneck events (Lin et al. 2014; Blanca et al. 2015; Razifard et al. 2020), which should increase the action of drift and decrease the efficiency by which deleterious mutations can be purged from populations. Additionally, tomato domestication has been accompanied by extensive selective sweeps (Razifard et al. 2020), which can lead to hitchhiking of deleterious alleles with positively selected variants. Lastly, modern cultivated tomato has drastically expanded its population size as humans spread it around the world, a situation that can lead to accumulation of deleterious alleles via ‘gene surfing’ (Klopfstein et al. 2006; Travis et al. 2007).

Our findings, however, suggest that the cost of domestication in tomatoes does not manifest as a simple greater number of deleterious mutations in domesticated genomes. In fact, we observed a downward trend throughout tomato domestication history in the average number of deleterious mutations per genome, with the highest levels occurring in wild SP and the lowest in modern SLL (Figure 1c). This is similar to what was observed for domesticated sorghum (Lozano et al. 2021) and soya bean (Kim et al. 2021) but has not been observed or expected for most domesticates. However, the trend of decrease in the absolute number of deleterious alleles through tomato domestication was concordant with that of all categories of mutations examined and for nucleotide diversity in tomato populations (Figure 1; Figure S2).

Our observations suggest that bottleneck events associated with the partial domestication of SLC and the more complete domestication of SLL have had a major impact on the absolute number of deleterious mutations during tomato domestication history. Additionally, SP outcrosses to a greater degree than SLC and SLL and has higher levels of heterozygosity (Razifard et al. 2020), which can mask the fitness effects of deleterious alleles. Thus, the transition to higher levels of self‐fertilisation, which leads to increased homozygosity and was further accelerated under human management of SLL, could have facilitated the purging of deleterious alleles (Byers and Waller 1999; Liu et al. 2017; Dwivedi et al. 2023) affecting the absolute number of deleterious mutations during tomato domestication.

Population bottlenecks remove many mutations from a population, regardless of their impact on fitness. Thus, the presence of bottlenecks can complicate cross‐population comparisons on the prevalence of deleterious mutations. We thus advocate for the use of ratios such as *nonsyn./syn*. and relative mutational load – which we define as the ratio of the number of sites with derived deleterious alleles to the number of sites with neutral nonsynonymous alleles (*del./neutr. nonsyn*.) – along with deleterious allele frequencies to provide more accurate comparisons of the mutational cost of domestication among populations. Similarly, studies in sunflower (Renaut and Rieseberg 2015), as well as sorghum and maize (Lozano et al. 2021), have shown that changes in the proportion of deleterious alleles may differ from changes in the absolute number of deleterious alleles when comparing domesticated populations against their wild relatives.

By focusing on these relative measures, we discovered different fates for deleterious mutations in each tomato population. Although SP accessions have the highest numbers of sites with derived deleterious alleles per genome (Figure 1c), our results show that wild SP harbours a relatively low *nonsyn./syn*. and relative mutational load (*del./neutr. nonsyn*.) compared to other populations (Figure 2a,b). In contrast, modern cultivated SLL harbours more relative mutational load compared to SP, confirming estimates by (Koenig et al. 2013), despite having a significantly smaller median per genome number of sites with derived deleterious alleles than SP. However, our results suggest that the largest changes in relative mutational load in the history of tomato domestication occurred during the divergence of SP and SLC in Ecuador (Figure 2a,b). This observation is further supported by the change in the proportion of nonsynonymous derived alleles. A more modest increase in relative mutational load compared to most South American SLC groups is also evident in the SLC populations that spread northward to Mesoamerica (SLC MEX and SLC MEX‐CA‐NSA) (Figure 2b), although the proportion of nonsynonymous mutations (Figure 2a) is higher in these populations compared to SLC ECU. This incongruence might suggest that the first northward spread of SLC might have entailed release from artificial selection pressure and/or positive selection on nonsynonymous substitutions that might have been beneficial for the survival of this population in more northern wild‐like habitats (Razifard et al. 2020).

Curiously, the SLL populations, which are thought to be derived from SLC MEX, retain similar or only modestly higher levels of relative mutational load compared to northern SLC populations, but without such high proportions of nonsynonymous mutations per genome. This implies that the origin of the commonly cultivated tomato in Mexico did not entail an increase in relative mutational load. However, we observed a very high variance in proportions of nonsynonymous and deleterious alleles among modern SLL accessions. Interestingly, the highest relative mutational loads among all groups were observed for SLC San Martin, a South American SLC group that displays more domestication‐like traits than other SLC groups (Razifard et al. 2020). Additionally, in both SLL groups and SLC San Martin, the frequencies of derived deleterious alleles are higher than in other groups (Figure 2d), suggesting that in all three groups a cost of domestication may be manifesting through the pervasiveness of the deleterious alleles that still persist.

Our observations altogether suggest that (a) drastic changes in proportions of nonsynonymous and deleterious derived alleles per genome have occurred, not during the final stage of tomato domestication, but rather with the origin of its semi‐domesticated relative, SLC; (b) many neutral or beneficial nonsynonymous mutations might have increased during the northward spread of SLC; (c) during modern breeding of the domesticated tomato, some neutral or beneficial nonsynonymous mutations may have been purged out of modern SLL, compared to its closest relative SLC MEX, although the proportion of its deleterious alleles has remained, more or less, the same; and (d) the derived deleterious alleles that do exist in the commonly cultivated tomato and SLC San Martin are more common than expected. Therefore, in modern cultivated tomato, the cost‐of‐domestication hypothesis (Lu et al. 2006) is manifested partially in loss of non‐deleterious alleles as well as gain in frequency among deleterious alleles. An important possibility to consider, but that cannot be assessed from our data, is that a subset of alleles identified as deleterious in our domesticated populations may in fact correspond to alleles favoured during breeding in some or all cultivars and thus account for the higher frequencies in these groups. In fact, several examples of loss of function mutations that on the surface would seem deleterious but have been favoured under domestication in crop systems exist (Monroe et al. 2020; Dwivedi et al. 2023). However, the prevalence of these types of domestication alleles across crops is not known.

The genetic cost of tomato domestication was also evident among private alleles. In all tomato populations, except SP, there is a higher proportion of deleterious alleles, compared to neutral nonsynonymous alleles, that are private to one population (not shared by any other population; Figure S3). Notably, most deleterious alleles found in the northern SLC populations as well as SLL seem to be private. Additionally, the private deleterious alleles are more common than expected in all SLC and SLL populations (Figure S4). These observations suggest that under domestication, the deleterious alleles probably have a recent origin, but become more common than in the wild. These observations also confirm that, although some deleterious alleles may be escaping selection, deleterious alleles overall are more efficiently purged out of wild tomato populations than those under domestication. Thus, an increase in mutational load during the origin of SLC and subsequent tomato domestication seems to be manifested more strongly in private alleles than those shared between populations.

Exploring genomic factors affecting the cost of tomato domestication, we found a correlation between mutational load and three genomic factors (Table 1). Overall, a higher mutational load was observed in genomic regions with high average nucleotide diversity, low recombination and weaker selective sweep signals. A study in soya bean (Sun, Wang, Li, et al. 2023) also reported a lower proportion of deleterious alleles in sweep regions. These observations might reflect an increased mutational load in genomic regions under relaxed selection. However, several factors might be involved in creating this pattern. Alternatively, genetic hitchhiking of deleterious alleles on beneficial alleles occurs in areas under ‘soft’ selective sweeps, where a considerable level of nucleotide diversity is still present in a population (McCoy and Akey 2017). In contrast to soft sweeps, ‘hard’ selective sweeps lead to greater loss of nucleotide diversity, impacting alleles from all fitness categories including deleterious alleles, in the genomic regions under strong or ancient selective sweeps.

Exceptions to the general patterns of association between mutational load and genomic factors were observed in SLC San Martin and the two SLL populations. This is interesting considering large average fruit sizes (Figure S1a) as well as low genetic diversity (Figure 1b) and higher frequency of deleterious alleles than expected (Figure 2c) in these populations. These observations imply that SLC San Martin may have undergone a demographic history comparable with that of the commonly cultivated tomato groups, which has led to the decoupling of deleterious allele accumulation and recombination rate.

### 4.2 The Evolution of Deleterious Load in the Context of Genomic Structural Variants

Our results revealed a reduction in the number of SVs through tomato domestication history (Figure 3a), which is similar to the pattern observed for the number of mutations from different categories, that is, non‐coding, synonymous, nonsynonymous and neutral nonsynonymous (Figure 1c,d and Figure S2). Such reduction can also be attributed to loss of overall genetic diversity (Figure 1b), due to bottleneck events, through tomato domestication history (Lin et al. 2014; Blanca et al. 2015; Razifard et al. 2020).

Furthermore, for most accessions, we discovered a significantly larger number of SVs in non‐genic regions of the tomato genome (Figure S5). This is consistent with the theory that SVs occurring in genic regions might have a strongly negative impact on the fitness of the individuals by disrupting gene function and thus will be removed from the population by selection (Vaughn et al. 2021). A study in Asian rice, *Oryza sativa* , also suggested that SVs are more likely to be deleterious (Kou et al. 2020). Among SVs in genic regions, most were found in introns and untranslated regions (Figure S6a), although when adjusting for the total size of intragenic features, we found SVs to be more prevalent in the 5′ and 3′ regions, with higher adjusted counts for exonic than intronic regions (Figure S6b). Together, these results suggest strong selection against SVs in genic regions, especially those within introns and exons. However, it is surprising to find larger adjusted numbers of SVs within exonic regions than intronic. Although this observation requires further analysis, one potential cause of this phenomenon could be linkage disequilibrium between SVs in the introns with variants under negative selection (Geibel et al. 2022).

Finally, we discovered a larger relative mutational load (*del*./*neutr. nonsyn*.) within SVs compared to genomic regions not impacted by SVs (Figure 3b). This observation implies that SVs might lead to gene disruption and pseudogenisation, which creates genomic hotspots for the accumulation of deleterious mutations. Alternatively, it is possible that previously pseudogenised genes harbour a larger number of SVs and deleterious mutations, due to relaxed selection.

### 4.3 Functional Link Between Deleterious Mutations and Desirable Traits for Tomato Breeding

Most methods for detecting deleterious mutations estimate the impact of a mutation based on the degree of the phylogenetic conservation of the gene in which that mutation occurs. This theory is valid for species living in similar environments but might be difficult to justify for closely related organisms that live in drastically different environments where a non‐conserved mutation may in fact be favourable (Tellier et al. 2011). This latter scenario is often the case for domesticated organisms, which live in a broad range of habitats compared to their wild relatives. Tracking the evolutionary trajectory of the deleterious alleles we discovered, we found that many of these have been purged from the commonly cultivated tomato (see Table S1), confirming the results discussed in previous sections. Focusing on the deleterious alleles that did persist in modern SLL, our GO‐term analyses revealed that these alleles impacted genes involving a broad spectrum of functions. Among these, genes involved in the ascorbic acid (vitamin C) pathway, which has an important role in the neutralisation of reactive oxygen species (Mellidou et al. 2012), and pollen germination under heat stress, were overrepresented among the GO terms. These GO terms suggest that some of the identified deleterious alleles could be in fact adaptations to a diverse range of habitats in the domesticated tomato.

Considering the importance of identifying potentially deleterious alleles for their possible impact in crop improvement, we created TomDel, a public repository of identified deleterious alleles present in the domesticated tomato and its closely related wild and semi‐wild populations. This repository, combined with other gene‐specific information on Solanaceae Genomic Network (https://solgenomics.net/), provides information on the probable population of origin as well as genotype frequencies of potentially deleterious alleles present in modern SLL and its closely related populations. Thus, it will serve as a guide for breeding purposes with the aim of avoiding the unintentional introduction of deleterious mutations to the tomato varieties of interest during experimental crossing, or for mining alleles identified as deleterious but that could be desirable for breeding purposes. For considerations on how the identification of potentially deleterious alleles can inform breeding practices, see Dwivedi et al. (2023).

Among well‐studied tomato genes, we found deleterious alleles with high frequency (> 50%) in genes involved in response to biotic and abiotic stress as well as fruit development and flavour regulation (Figure 4, Table 2). Notably, we found a potentially deleterious allele in *SlAAT4*, a known gene involved in the biosynthesis of fruit volatiles, which are essential for fruit flavour. This motivated us to conduct GWAS on several volatiles to search for deleterious alleles that might be associated with fruit volatile biosynthesis.

Our GWAS results (Figure 5a,b) revealed potentially deleterious alleles associated with methyl salicylate and isobutyl acetate concentrations in tomato fruits. In previous studies, no significant correlation has been found between methyl salicylate and flavour preference (Tieman et al. 2017). However, methyl salicylate is a well‐studied compound involved in disease resistance in plants; for example, see Min et al. (2018), as well as regulating flowering time (Banday and Nandi 2015).

The deleterious allele associated with methyl salicylate is within *STM3*, which codes for a transcription factor, putatively involved in regulating flowering time (Jiang et al. unpublished data) and inflorescence branching (Alonge et al. 2020) in tomato. This deleterious allele is negatively correlated with methyl salicylate concentrations and it is only found in SLL Americas. Therefore, it is likely that this deleterious allele arose during the origin of SLL but was selected against during the modern breeding of the commonly cultivated tomato.

Our results also revealed a derived deleterious allele associated with isobutyl acetate concentrations in tomato fruits (Figure 5b). Isobutyl acetate was previously reported to be negatively correlated with flavour preference in tomato, ‘overall liking’ (Tieman et al. 2017). This deleterious allele is within *Solyc01g108480*, which codes for a serine carboxypeptidase, suggested to be involved in response to wounding in tomato leaves (Moura et al. 2001). The absence of this allele in SLC San Martin, northern populations of SLC and both SLL populations is consistent with selection for lower levels of isobutyl acetate during tomato domestication to improve flavour preference. This finding suggests that selection mediated by humans against an allele identified as deleterious, which might nevertheless have an important function in the wild, considering how frequently it occurs in SP and its possible association with defence. This illustrates how it is crucial to consider the environment as well as the prevalence of the alleles identified as deleterious to better estimate the fitness effect of the allele.

## 5 Conclusions

In this study, we generated novel insights into the evolution of deleterious mutations through tomato domestication history, especially in the context of demographic factors as well as genomic structural variants (SVs). Our results help elucidate the overall dynamics of deleterious mutations as well as within different stages of tomato domestication, that is, initial domestication in Ecuador, northward spread of SLC towards Central America and Mexico, and re‐domestication of SLL in Mexico. Our results also revealed novel insight on the genomic factors, such as SVs and recombination rate, involved in shaping the deleterious load through tomato domestication history.

Through this study, we also attempted to create a functional link between mutations identified as deleterious and the phenotypes impacted by such mutations. In many functional genetic studies, the causal allele underlying a phenotype is difficult to pinpoint even after identifying the causal gene. Our approach helps narrow down the list of candidate alleles by identifying those with a potentially high impact on the phenotype of interest. Our findings can also be used for guiding breeding experiments. Using our results, breeders can avoid accidentally introducing potentially deleterious alleles into the tomato variety of interest from its closely related populations.

As our results revealed for isobutyl acetate, some mutations identified as deleterious by currently available methods might in fact confer an important function in the wild. Therefore, phrases such as ‘deleterious mutations’ and ‘mutational load’ might not fully capture the diverse functional impacts of mutations with high impacts on underlying phenotypes. In fact, there are some examples of loss of function mutations that seem to confer human‐desired traits in crops (reviewed by Monroe et al. 2020; Dwivedi et al. 2023). Accordingly, our uses of such phrases throughout this paper refer to high‐impact mutations identified as deleterious by two commonly used algorithms. We acknowledge that some of the ‘deleterious’ mutations reported in this study might confer desirable traits in domesticated organisms.

## Author Contributions

H.R. planned the research, conducted data analysis, developed the TomDel database and wrote the manuscript. S.V. contributed to structural variant detection and provided feedback on the manuscript. N.M. and L.M. hosted TomDel on the Sol Genomics Network and incorporated the new results with existing resources on tomato genes. D.T. contributed volatile data to genome‐wide association studies (GWAS) and provided feedback on genes associated with each GWAS signal. E.K. contributed phenotypic data to GWAS and provided feedback on the manuscript. A.L.C. provided feedback on all of the analyses throughout the study, provided feedback on the manuscript and edited the manuscript. All authors have read and approved the final manuscript.

## Conflicts of Interest

The authors declare no conflicts of interest.

## Supporting information


Figures S1–S6.

Table S1.

Table S2.

Table S3.

Table S4.

Table S5.

Table S6.

## Data Availability

The results of the analyses involving detection of deleterious alleles were provided as a public repository, called TomDel, available from https://github.com/hrazif/TomDel‐0.1. All genomic data included in this study are available from NCBI Short Read Archive, under SRP150040. Novel custom scripts created for this study were deposited into Github (https://github.com/hrazif/scripts_for_tomato_deleterious_mutations_paper). Benefit Sharing Statement: Benefits Generated: Benefits from this research accrue from the sharing of our data and results on public databases as described above.
