# Supplementary material for: The Evolutionary Dynamics of Genetic Mutational Load Throughout Tomato Domestication History
Source: Mol Ecol. 2025 Jul 15;34(24):e70024. doi: 10.1111/mec.70024 (PMC12717970; doi:10.1111/mec.70024)
Supplement: Supplementary file 1 — Figures S1–S6. [file MEC-34-e70024-s001.docx]

Supplemental Figure 1. Tomato population phylogeny and geographic distribution. (**a**) A maximum-likelihood phylogeny reconstructed using TreeMix. All nodes in the population tree received bootstrap support of 100%. (**b**) Distribution map modified from Razifard et al. [42] of all tomato accessions included in this study, except SLL Modern, which have a worldwide distribution
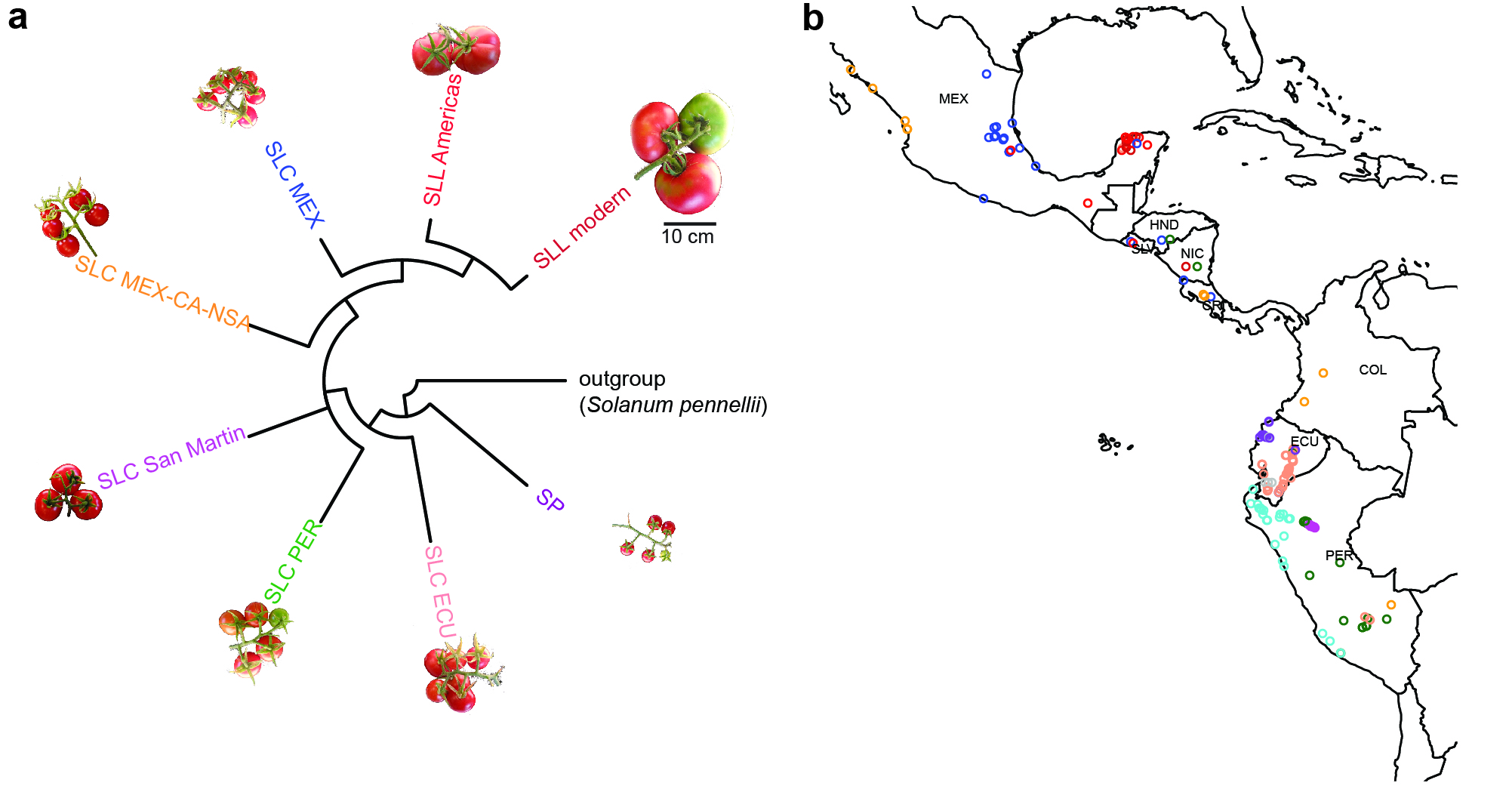
.


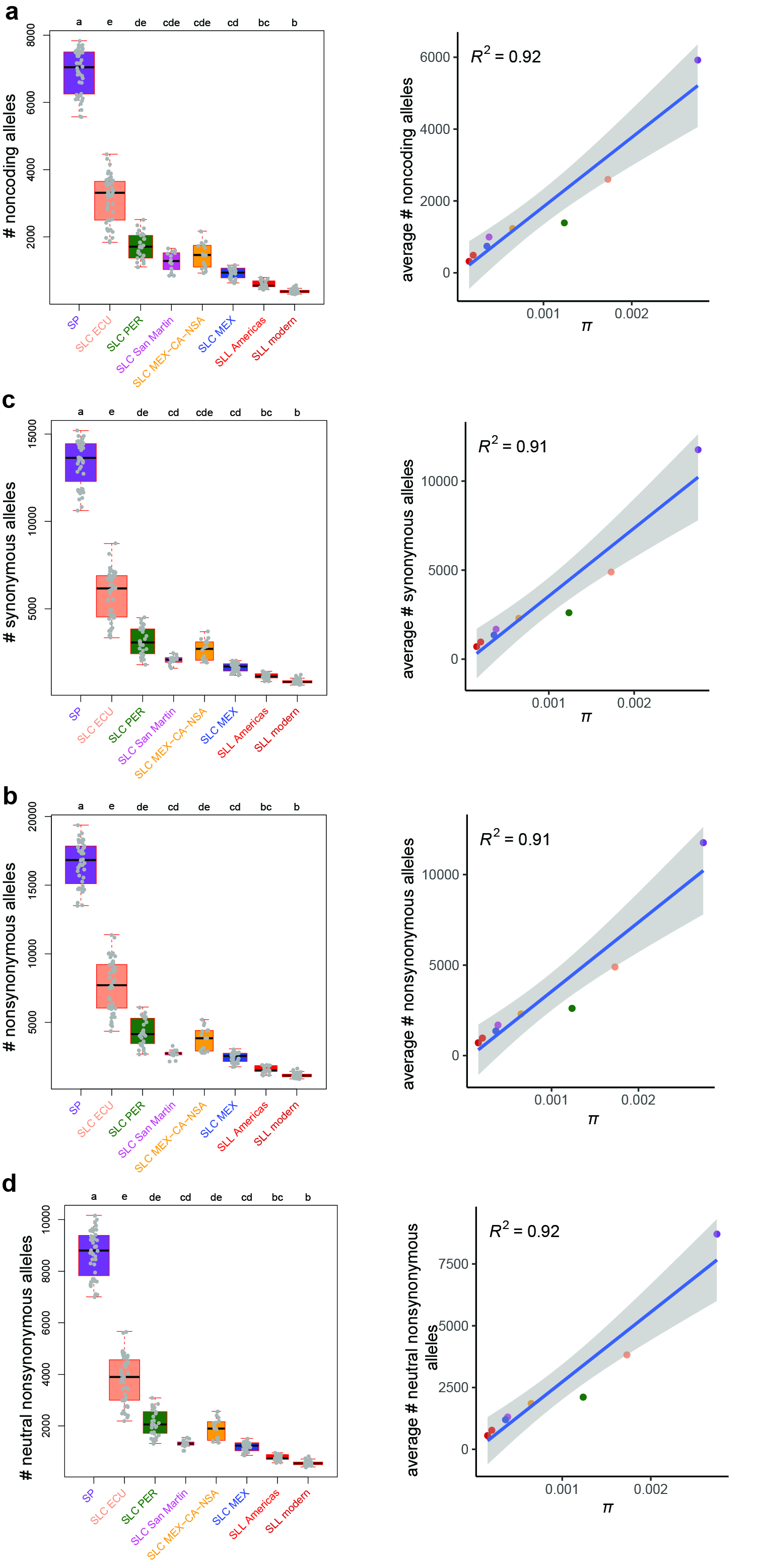


Supplementary Figure 2. The counts of sites with derived alleles in in different categories and their correlation with genetic diversity (*π*), showing a) a downward trend when comparing SLC populations with SP and SLLs with SLC; b) significant correlation between *π* and the per-population average number of alleles from all categories examined. Statistically non-significant comparisons, according to Dunn's test, between the populations in terms of the absolute number of alleles have been indicated using lower-case numbers.


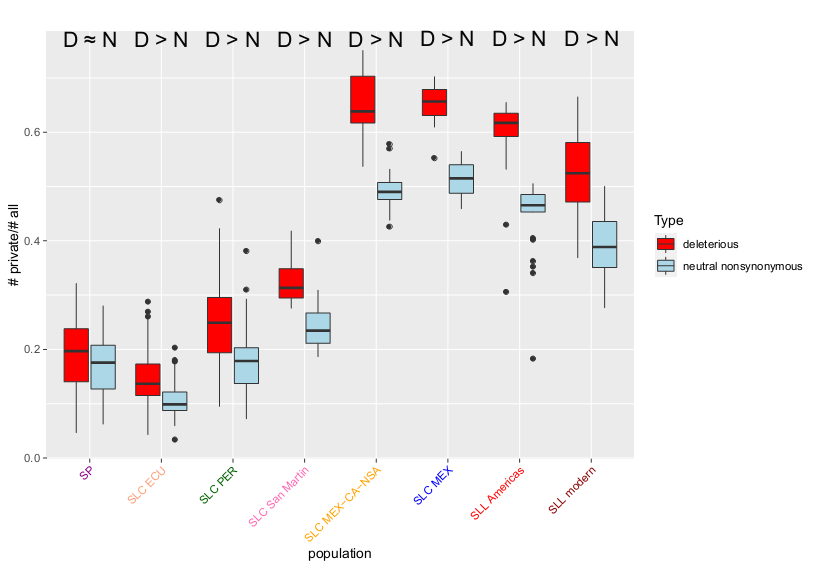


Supplemental Figure 3. Per-accession proportion of private derived deleterious to all derived deleterious (red) and private derived neutral nonsynonymous to all derived neutral nonsynonymous (blue) mutations. For each population, a significant t-test result (Bonferroni-adjusted p-value < 0.05) is shown with D > N, i.e. the ratio of private derived deleterious alleles to all derived deleterious is higher than the ratio of private derived neutral nonsynonymous alleles to all derived neutral nonsynonymous alleles. No significant different was observed for SP (D ≈ N).


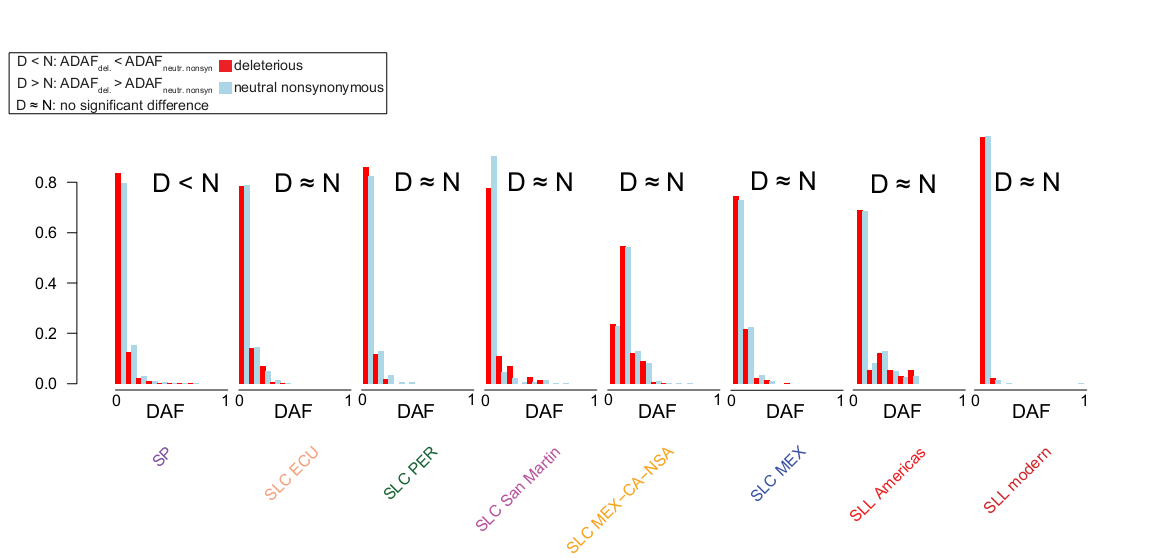


Supplemental Figure 4. Site frequency spectra for private deleterious (red) and private neutral nonsynonymous (blue) mutations in different tomato populations. Results of statistical tests of average derived allele frequency of deleterious alleles (ADAF_del_) versus average derived allele frequency of neutral nonsynonymous alleles (ADAF_neutr. nonsyn._) are shown with D < N (ADAF_del_ is smaller than ADAF_neutr. nonsyn._), D > N (ADAF_del_ is greater than ADAF_neutr. nonsyn._ l), and D ≈ N (ADAF_del_ is not significantly different from ADAF_neutr. nonsyn._).


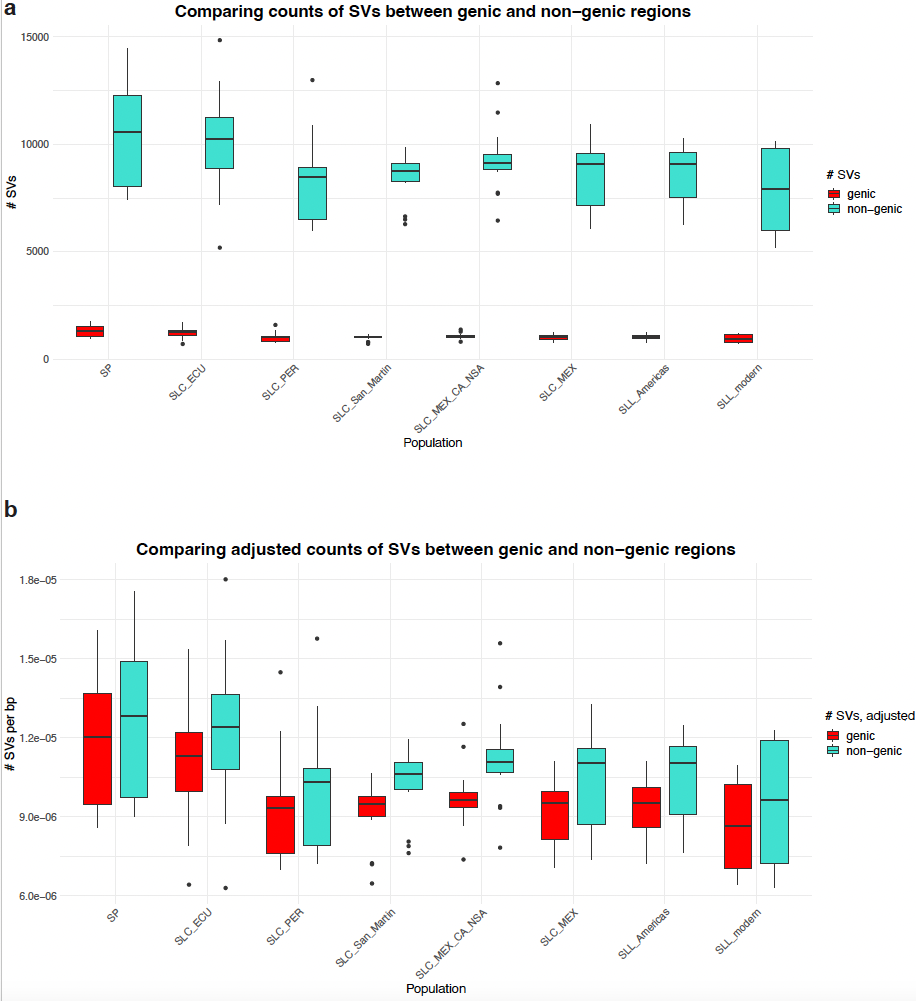
Supplemental Figure 5. A genome-wide comparison of the number of SVs within or outside genic regions in each tomato population. **a:** number of SVs in genic and non-genic regions; **b**: same as **a** but SV numbers adjusted for the total size of the genic and non-genic regions (109,855,134 and 714,088,907 bp, respectively). Comparisons within all populations in **a** and **b** are statistically significant(based a T-test; p-value < 0.001).


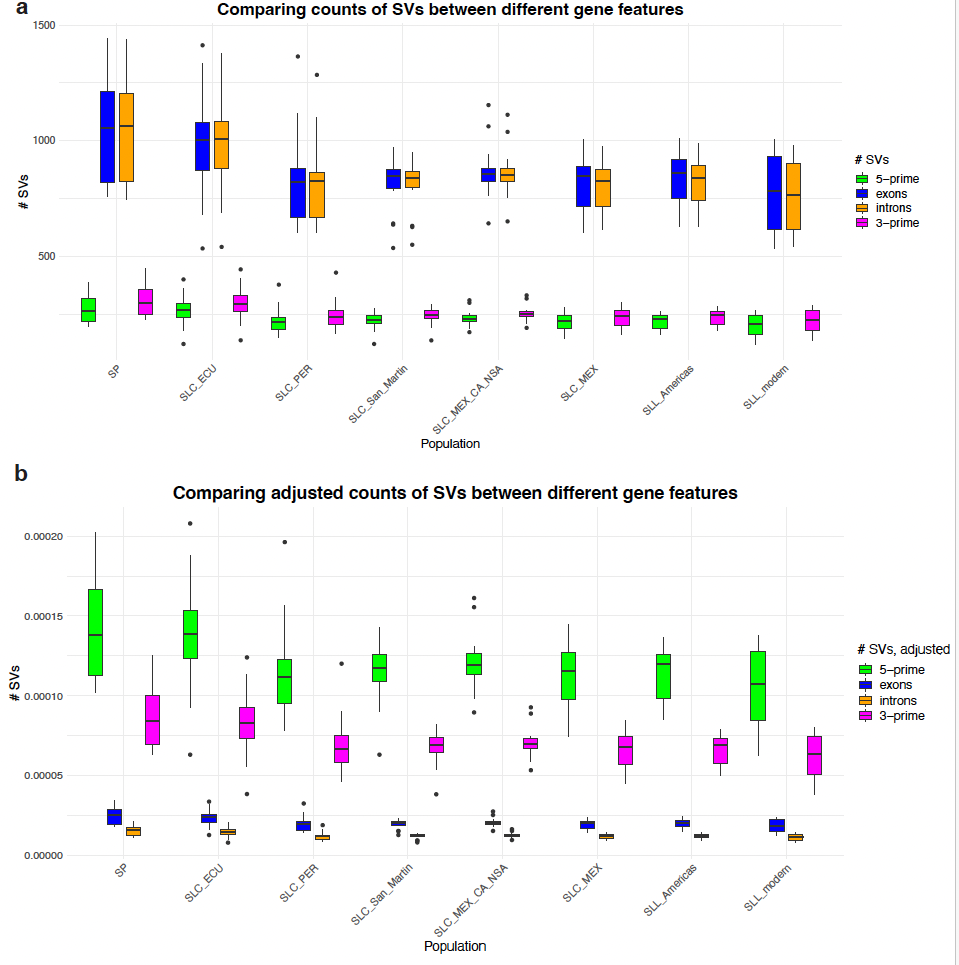


Supplemental Figure 6. A genome-wide comparison of the number of SVs within 5’ UTRS, introns, exons, and 3’ UTRs in each tomato population.

**a:** number of SVs within each gene features; **b**: same as **a** but SV numbers adjusted for the total genomic size of the five-prime (1,926,171 bp), intron (67,873,566 bp), exons (41,981,568 bp), and three-prime regions (3,580,357 bp).
